# The effect of ketamine on the consolidation and extinction of contextual fear memory

**DOI:** 10.1177/0269881117748903

**Published:** 2018-01-17

**Authors:** Nicholas E Clifton, Kerrie L Thomas, Jeremy Hall

**Affiliations:** 1 Neuroscience and Mental Health Research Institute, Cardiff University, Cardiff, UK; 2 School of Biosciences, Cardiff University, Cardiff, UK

**Keywords:** Contextual fear conditioning, ketamine, extinction learning, schizophrenia, *N*-methyl-ᴅ-aspartate receptor

## Abstract

Ketamine, principally an antagonist of *N*-methyl-ᴅ-aspartate receptors, induces schizophrenia-like symptoms in adult humans, warranting its use in the investigation of psychosis-related phenotypes in animal models. Genomic studies further implicate *N*-methyl-ᴅ-aspartate receptor-mediated processes in schizophrenia pathology, together with more broadly-defined synaptic plasticity and associative learning processes. Strong pathophysiological links have been demonstrated between fear learning and psychiatric disorders such as schizophrenia. To further investigate the impact of ketamine on associative fear learning, we studied the effects of pre- and post-training ketamine on the consolidation and extinction of contextual fear memory in rats. Administration of 25 mg/kg ketamine prior to fear conditioning did not affect consolidation when potentially confounding effects of state dependency were controlled for. Pre-training ketamine (25 mg/kg) impaired the extinction of the conditioned fear response, which was mirrored with the use of a lower dose (8 mg/kg). Post-training ketamine (25 mg/kg) had no effect on the consolidation or extinction of conditioned fear. These observations implicate processes relating to the extinction of contextual fear memory in the manifestation of ketamine-induced phenotypes, and are consistent with existing hypotheses surrounding abnormal associative learning in schizophrenia.

## Introduction

Ketamine has been the focus of much research in psychiatry due to its psychotomimetic effects in healthy humans and the fact that it has been shown to augment psychotic symptoms in patient populations (Corlett et al., 2007b, 2016; Javitt and Zukin, 1991; Krystal et al., 1994; Newcomer et al., 1999). Among several off-site targets (Sleigh et al., 2014; Zanos et al., 2016), ketamine principally acts as an antagonist of *N*-methyl-ᴅ-aspartate (NMDA) glutamate receptors, which have important roles in synaptic plasticity processes (Lüscher and Malenka, 2012; Riedel et al., 2003). The importance of altered NMDA receptor complex function in psychotic disorders is further highlighted by studies showing that mutations from patients with schizophrenia are enriched in genes encoding components of NMDA receptor signalling complexes, as well as genes involved in synaptic plasticity, associative learning and memory (Fromer et al., 2014; Kirov et al., 2012; Pocklington et al., 2015; Purcell et al., 2014; Ripke et al., 2014). Investigating the behavioural effects of ketamine on basic learning processes is thus important for understanding how altered NMDA receptor function may contribute to the pathogenesis of psychosis.

Altered associative learning and related plasticity processes have been linked to schizophrenia through behavioural and genomic studies (Diwadkar et al., 2008; Pocklington et al., 2015; Ripke et al., 2014) and have been proposed to contribute to the manifestation of positive symptoms (Corlett et al., 2009; Fletcher and Frith, 2009; Hall et al., 2009; Martins Serra et al., 2001). Fear conditioning is an evolutionarily-conserved form of associative learning, making it a suitable phenomenon for studying in model organisms in the investigation of the regulation and dysregulation of aversive associative memories (Milad and Quirk, 2012; Pattwell et al., 2012). Distinct component processes of learning can be investigated, including associative fear memory consolidation and extinction (Lee et al., 2004; Pedreira and Maldonado, 2003). Consolidation is the time-delimited process that stabilizes an association between a neutral stimulus (conditioning stimulus (CS)) and an unconditioned stimulus (US), such as an aversive footshock, presented contiguously, so that presentation of the CS elicits a conditioned fear response. Extinction of conditioned fear occurs when the subject is re-exposed to the CS in the absence of the US for an extended period or repeatedly. Extinction results in the loss of the conditioned response. Both the consolidation and extinction of a fear memory are considered forms of new learning (Ochs, 1968), yet relate to behaviourally antagonistic components of Pavlovian conditioning (Bouton, 1993; Pavlov, 1927; Rescorla and Heth, 1975). Analysis of genomic data has recently highlighted a particular impact of schizophrenia-related copy number variants on molecular processes underlying fear extinction (Clifton et al., 2017), in line with previous behavioural studies suggesting abnormalities of inhibitory learning in schizophrenia (Holt et al., 2009; Martins Serra et al., 2001; Millan et al., 2012; Pocklington et al., 2015).

Ketamine has been used in many studies to model schizophrenia-like symptoms in rodents and as a tool for the identification of potential therapeutics (Castner et al., 2010; Chan et al., 2012; Frohlich and Van Horn, 2014; Nikiforuk and Popik, 2012; Nikiforuk et al., 2010; Pitsikas and Markou, 2014). Previous investigations into the effects of ketamine on components of aversive associative learning have principally been directed at cued tone-shock, amygdala-mediated learning, rather than single trial contextual fear conditioning mediated by the hippocampus. Furthermore, a number of apparent discrepancies exist between the results of these studies. Using rats, Pietersen et al. (2006) found that pre-training ketamine (16 mg/kg) blocked fear conditioning to a tone-shock CS-US pairing (Pietersen et al., 2006). Conversely, others have found that pre-training ketamine (8 mg/kg) or post-training with high doses (four injections of 100/50/50/50 mg/kg spaced at 60 min intervals) had no effect on cued fear conditioning (Bolton et al., 2012; Groeber Travis et al., 2015). Bolton et al. (2012) also reported that ketamine (8 mg/kg) had no significant effect on contextual fear conditioning.

The hippocampus plays a key role in contextual fear conditioning, interacting with the amygdala, pre-limbic and infralimbic cortical areas (Hugues and Garcia, 2007; Ji and Maren, 2007; Radulovic and Tronson, 2010; Tronson et al., 2012). Contextual associative learning is critical for faithful information retrieval and logical inference (Maren et al., 2013) and a breakdown in these processes may contribute to the development of psychotic symptoms. Indeed, substantial evidence implicates impaired contextual processing and altered hippocampal function in schizophrenia (Holt et al., 2012; Maren et al., 2013; Matosin et al., 2016; Taylor et al., 2012).

To further investigate the impact of ketamine on contextual associative learning, we measured the effects of pre- and post-training ketamine (8 mg/kg and 25 mg/kg) on the consolidation and extinction of contextual fear memories in rats to determine the sensitivity of excitatory and inhibitory associations, respectively, with ketamine.

## Materials and methods

### Subjects

Subjects were 80 adult male Lister Hooded rats (Charles River, UK) weighing 275–325 g, naïve to testing or drug administration. Rats were housed in pairs in conventional NKP RC2R cages, each containing wood shavings, bedding and a cardboard tube for environmental enrichment, within a holding room maintained at 21°C on a 12-hour reversed light/dark cycle (lights on 20:00) and with food (Harlan 2014 global rodent diet) and water access ad libitum. All experiments were performed during the dark phase of the cycle. Rats were left to acclimatise for at least five days before testing. The handling of animals from each experimental group was ordered pseudorandomly. All procedures were conducted in accordance with local Cardiff University Ethical Committee approval and the UK 1986 Animals (Scientific Procedures) Act (Project license PPLs 30/2236 and 30/2722).

### Contextual fear conditioning

The contextual fear conditioning protocol described herein is a well-established procedure for studying associative memory in rodents (Barnes and Thomas, 2008; Lee et al., 2004). Rats were individually exposed to a novel context for a three-minute conditioning training trial, during which they received a single scrambled footshock (0.5 mA for two seconds) two minutes after being placed into the novel context. Rats were then returned to home cages. Memory was assessed in up to three subsequent recall trials at two days (Recall 1), four days (Recall 2) and seven days (Recall 3) post-conditioning, during which rats were re-exposed to the same context. In recall trials re-exposure was for two minutes (insufficient to produce extinction (Barnes and Thomas, 2008)). In extinction experiments, rats underwent 10 min re-exposure to the context two days after conditioning. Following extinction training, recall trials were two days (Recall 1) and five days (Recall 2) afterwards. Freezing behaviour was recorded as an index of conditioned fear during conditioning and recall trials. Freezing was quantified as a proportion of total time, and defined as the cessation of movement within a one-second period, sampled manually every 10 s, consistent with previous studies (Barnes et al., 2012; Barnes and Thomas, 2008; Trent et al., 2015).

To assess the effect of 25 mg/kg ketamine on contextual fear memory consolidation, rats (*n*=6; Table 1 in Supplementary Material) were treated with ketamine 30 min before conditioning and Recall 2. Controls animals (*n*=6) were treated with saline before the same trials. When testing the effect of 8 mg/kg ketamine on fear memory consolidation, one group (*n*=4) received ketamine 30 min before conditioning and saline before Recall 1 whilst a second group (*n*=4) was treated with ketamine prior to both trials.

In extinction experiments, two groups (*n*=6 per group) received 25 mg/kg ketamine 30 min before extinction training, of which one group was treated with ketamine before conditioning and one group received saline before conditioning. Prior to Recall 1 and Recall 2, these groups received the same treatment, or opposite treatment, to that given before conditioning, respectively, in order to control for state-dependent effects of the drug on the conditioned memory. Control subjects (*n*=6) received saline prior to all trials.

In low-dose extinction experiments, 8 mg/kg ketamine or saline was administered 30 min prior to extinction training. Each treatment group was subdivided into two groups (*n*=6), which received 8 mg/kg ketamine or saline prior to Recall 1.

In post-training ketamine experiments, rats (*n*=6 per group) received 25 mg/kg ketamine or saline immediately upon removal from the context, following conditioning and extinction. Control subjects received saline after each trial. All groups received saline after Recall 1.

### Drugs

Ketamine hydrochloride (Ketaset, Henry Schein Animal Health, UK) was diluted in a normal saline vehicle to 25 mg/mL or 8 mg/mL and administered intraperitoneally (IP) at 1 mL/kg 30 min prior or immediately following conditioning training or recall trials. The resulting subanaesthetic doses of 25 mg/kg or 8 mg/kg are consistent with previous studies (Bolton et al., 2012; Razoux et al., 2007).

### Statistical analysis

Percentage time spent freezing was normalised to the post-shock (post-US) period, thereby expressing freezing behaviour relative to subject response to US and reducing the effect of non-specific inter-subject variability. Post-US freezing of each subject was expressed as a percentage of the group mean, to maintain representation of the standard error. Within-trial effects were determined using two-way repeated measures (RM) analysis of variance (ANOVA). Post-hoc testing was subjected to Sidak’s correction method. Treatment effects in recall trials were determined using two-tailed Student’s *t*-tests for comparisons of two groups, or one-way ANOVA followed by Tukey’s multiple comparisons test for comparisons of more than two groups. For extinction experiments, treatment effects were analysed following the grouping of subjects by pre-extinction treatment. Significant group differences were identified using an alpha level of 0.05.

## Results

### Contextual fear conditioning following ketamine administration

**Figure 1.**
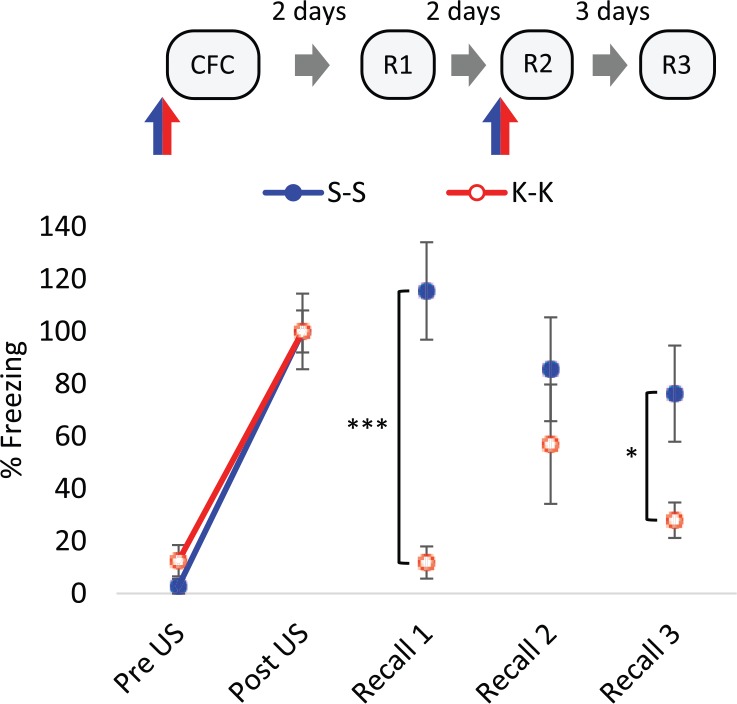
The effect of ketamine (K) on contextual fear conditioning (CFC). 25 mg/kg K induced a state-dependent contextual fear memory. K (25 mg/kg) or saline (S) vehicle were administered intraperitoneally (IP) 30 min before conditioning and Recall 2 (R2), indicated by blue/red arrows, *n*=6. Data represented by mean±standard error of the mean (SEM) of percentage freezing response normalised to post-unconditioned stimulus (post-US). ***p<0.001, *p<0.05 in Student’s *t*-test. R1: Recall 1; R3: Recall 3.

**Figure 2.**
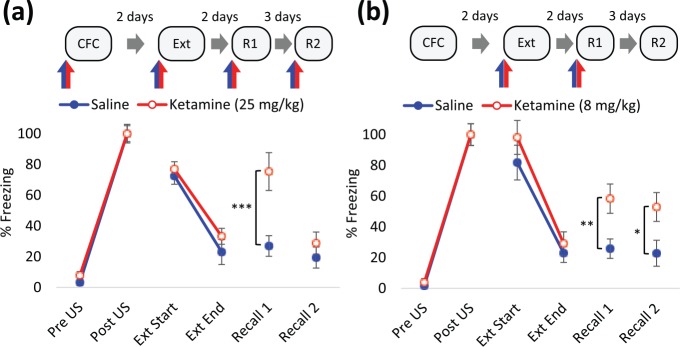
Ketamine impaired the extinction of contextual fear memory. Displayed are the first (extinction trial (Ext) Start) and last (Ext End) two minutes of the Ext (10 min). (a) 25 mg/kg ketamine or saline vehicle was administered intraperitoneally (IP) 30 min before trials, *n*=6 (saline) or 12 (ketamine) per group. Rats in the ketamine pre-extinction group received either saline or ketamine prior to conditioning. In recall trials, ketamine-treated rats received either the same (Recall 1 (R1)) or different (Recall 2 (R2)) administration to that received prior to conditioning, to control for state-dependent effects (expanded in Figure 1 in Supplementary Material). (b) 8 mg/kg ketamine or saline vehicle was administered IP 30 min before extinction training and R1, as indicated by blue/red arrows. *n*=12 per group. Rats in the ketamine group received either saline or ketamine prior to R1 to control for any state-dependent effects. (a) and (b) Data represented by mean±standard error of the mean (SEM) of percentage freezing response normalised to post-unconditioned stimulus (post-US). Data is grouped by pre-extinction saline- and ketamine-treatment. ***p<0.001, **p<0.01, *p<0.05 in Student’s *t*-test. CFC: contextual fear conditioning.

There was no effect of pre-training 25 mg/kg ketamine on freezing response pre- and post-shock (US) during the conditioning trial compared to saline control rats (*F*_(1,10)_=0.43, *p*=0.53, two-way RM ANOVA; Figure 1, Table 2 in Supplementary Material). Thus, ketamine had no effect on the response to context or the US and both saline- and ketamine-treated groups showed increases in freezing behaviour after brief presentation of a footshock (*F*_(1,10)_=82.78, *p*<0.001, two-way RM ANOVA). This indicates that ketamine had no non-specific effects on stimulus responsiveness and ketamine-treated rats could acquire a contextual fear memory similarly to control subjects. In a recall test, 48 h later, rats treated with 25 mg/kg ketamine before conditioning displayed substantially less conditioned freezing than saline controls (Figure 1). The implication from this finding is that ketamine prevents the stabilisation or consolidation of conditioned fear memory. However, the administration of ketamine prior to a subsequent recall test reinstated fear responding, such that there was a Recall×Treatment interaction from Recall 1 to Recall 2 (*F*_(1,10)_=8.70, *p*<0.05, two-way RM ANOVA) and the freezing response of ketamine-treated rats was not different from control levels (Figure 1). This suggests that ketamine acts to aid retrieval such that expression of the conditioned fear memory is dependent on the presence of both external and internal (interoceptive) cues. Evidence for this state-dependent retrieval of conditioned fear memory is shown in a further recall test (Recall 3) in the absence of ketamine, in which rats conditioned under ketamine displayed decreased freezing responses compared to controls once more (Figure 1). It should be noted that whilst the experiment was well powered for the observation of these group differences in freezing response, it is more difficult to conclude with confidence the absence of group differences (i.e. Recall 2). However, the retrieval of conditioned response following 25 mg/kg ketamine administration was demonstrated again subsequently (Figure 2).

### Extinction learning following ketamine administration

To assess the effect of ketamine on the extinction of fear memory, rats underwent contextual fear conditioning followed by an extinction trial 48 h afterwards, treated with 25 mg/kg ketamine or saline vehicle 30 min before each. The ketamine challenge prior to conditioning and extinction in the experimental group was to control for, and test for, state-dependent effects, respectively. Thus, training and testing of the fear responses occurs when the rats are in one interoceptive state. This is particularly important for the expression of extinction at recall, which depends on the retrieval of the CS-US and CS-no US memory (Bouton, 1993). In the 10-minute extinction trial, there was no treatment effect on conditioned responses during the first two minutes (*F*_(2,15)_=0.466, *p*=0.64, one-way ANOVA, Figure 1 in Supplementary Material). Hence, ketamine did not affect the recall of fear memory irrespective of pre-conditioning treatment. Within-session extinction was not influenced by treatment (*F*_(1,16)_=1.26, *p*=0.28, two-way RM ANOVA). In a subsequent recall trial, rats treated with saline prior to extinction exhibited reduced freezing responses, indicative of the loss of the conditioned response by extinction training (*p*<0.01, paired *t*-test; Figure 2, Table 3 in Supplementary). However, administration of ketamine before extinction attenuated the loss of conditioned response at Recall 2 (Figure 2). This could be due to inhibition of consolidation of the extinction memory by ketamine. The alternative interpretation is a state-dependency for the retrieval of the extinction memory. This explanation is discounted since the conditioned response is maintained in a group that received ketamine prior to both extinction training and Recall 1 (K-K-K-S, Figure 1 in Supplementary Material). In the absence of ketamine before recall, rats conditioned under ketamine displayed low levels of conditioned response (K-K-K-S Recall 2, Figure 1 in Supplementary Material), consistent with the idea that different interoceptive contexts disrupt the retrieval of the CS-US association.

In summary, our results show that ketamine administration prior to extinction impairs the consolidation of the CS to no-US association.

### Fear memory processing following administration of a lower dose of ketamine

Our data indicating that ketamine has a selective effect on the consolidation of extinction may be confounded by the state-dependency of the CS-US retrieval. To reduce the effect of this confound, we performed two further experiments: first, we reduced the dose of ketamine and secondly, we conducted fear conditioning and extinction training in the absence of ketamine, with its administration immediately after each trial, upon the removal of rats from the context.

In a pilot experiment, rats were conditioned following the administration of 8 mg/kg ketamine and were given a recall trial 48 h later following either 8 mg/kg ketamine or vehicle. During the recall trial, there was no effect of treatment on the freezing response (Figure 2 in Supplementary Material), suggesting that this dose of ketamine did not generate a state-dependent contextual fear memory, and retrieval was likely solely dependent on exposure to the physical context.

Similar to our previous result with a higher dose of ketamine, administration of ketamine at 8 mg/kg prior to extinction training had no effect on within session extinction *(F*_(1,22)_=0.98, *p*=0.33, two-way RM ANOVA) but attenuated the effect of extinction training on conditioned responses at Recall 1 (Figure 2). Note that this effect was observed irrespective of whether ketamine or saline was administered prior to testing (*F*_(1,20)_=0.01, *p*=0.91, two-way ANOVA) and therefore concurs with our pilot data that retrieval of the CS-US association is not dependent on the interoceptive status of the rat at recall. In a subsequent recall test, in the absence of treatment, an elevated freezing response in ketamine-treated rats remained.

Thus, ketamine selectively impairs the formation of the extinction memory whether conditioning occurred in the presence (to control for state-dependent retrieval) or absence of ketamine.

### Post-conditioning and post-extinction ketamine treatment

**Figure 3.**
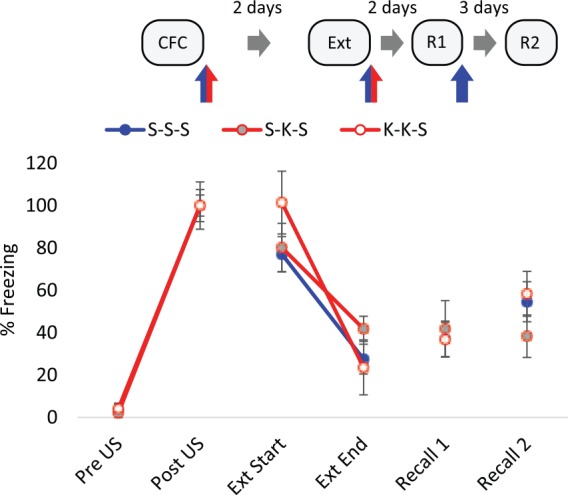
Post-trial administration of 25 mg/kg ketamine (K) had no effect on the consolidation or extinction of contextual fear memory. Displayed are the first (extinction trial (Ext) Start) and last (Ext End) two minutes of the Ext. K (25 mg/kg) or saline (S) vehicle was administered intraperitoneally (IP) immediately after conditioning, extinction and Recall 1 (R1), *n*=6 per group. Data represented by mean±standard error of the mean (SEM) of percentage freezing response normalised to post-unconditioned stimulus (post-US). CFC: contextual fear conditioning; R2: Recall 2.

Post-conditioning ketamine (25 mg/kg) had no effect on freezing during the first two minutes of the long recall trial (Figure 3), indicating that there was no effect of the drug on consolidation of the CS-US association. The extinction trial resulted in decreased within-trial freezing responses in all treatment groups (*F*_(1,15)_=53.62, *p*<0.001, RM ANOVA). Post-extinction ketamine had no effect on total freezing during subsequent recall trials (Figure 3), indicating no disruption to the consolidation of extinction memory. This shows that ketamine only exerts its effects on extinction memory when administered prior to training.

## Discussion

Our results indicate that ketamine impairs between-session extinction of contextual fear memory, but leaves within-session extinction and fear memory conditioning intact.

We have observed that systemic administration of the psychotomimetic, ketamine, prior to consolidation generates a state-dependent fear memory at 25 mg/kg – that is, rats were better able to recall association information about the context when they were subjected to the same internal context to which the information was acquired in. When this state-dependency is accounted for, we found that pre-training ketamine, at 25 mg/kg or 8 mg/kg, has no effect on contextual fear memory consolidation but diminishes the extinction effect of prolonged re-exposure to a contextual CS.

Ketamine did not affect the conditioned response when administered before or immediately after contextual fear conditioning training, indicating that the treatment had no effect on the acquisition or consolidation of the associative memory. This is consistent with the small amount of existing literature concerning the effect of ketamine on contextual fear conditioning (Bolton et al., 2012) and parallels some findings from cued fear learning (Bolton et al., 2012; Groeber Travis et al., 2015; but see Pietersen et al., 2006). However, inconsistencies in dose, protocol and the potential for state-dependency confound direct comparison across studies. Furthermore, chronic ketamine administration may impair the formation of contextual fear memories in rodents (Amann et al., 2009), although it is important to note that a single administration of ketamine is sufficient to induce temporary schizophrenia-like phenotypes in humans (Javitt and Zukin, 1991; Krystal et al., 1994; Newcomer et al., 1999).

We show that ketamine selectively impairs the extinction of contextual fear memory. Pre-extinction ketamine, at both doses, led to the maintenance of conditioned response even when ketamine was re-administered prior to the recall trial, indicating that this effect cannot be explained by state-dependency of the extinction memory. This finding parallels previous reports that NMDA receptor activation is required for fear extinction (Baker and Azorlosa, 1996; Burgos-Robles et al., 2007; Lee et al., 2006; Liu et al., 2009; Santini et al., 2001; Sotres-Bayon et al., 2007) and suggests that ketamine impairs specific component processes of associative learning. Ketamine at 25 mg/kg or 8 mg/kg did not influence within-session freezing response during the long recall trial, suggesting that ketamine has no effect on the acquisition of extinction learning and instead mediates its effects by interfering with the induction of molecular processes responsible for the subsequent consolidation of extinction memory. However, administration of ketamine immediately after extinction training did not induce the same deficit. Therefore, extinction memory was only impaired when ketamine was present during the acquisition of extinction. This is contrary to a report of impaired cued fear memory extinction by post-training administration of phencyclidine (PCP) (Pollard et al., 2012), perhaps reflecting mechanistic or off-site target differences between these NMDA receptor antagonists and psychotomimetics, or the involvement of NMDA receptors during predominantly hippocampus- or amygdala-mediated inhibitory learning.

Whilst this is, to our knowledge, the first time that ketamine has been shown to selectively impair the extinction of contextual fear memory, the same 25 mg/kg dose of ketamine has been found to impair inhibitory fear learning in another study. Razoux et al. (2007) reported that ketamine abolished the latent inhibition of conditioned fear (Razoux et al., 2007), referring to the weakening of the CS-US association due to extensive pre-exposure to the CS in the absence of the US, before conditioning (Westbrook and Bouton, 2010). Together, these results lend to the postulation that ketamine may have a selective effect on associative processes which modulate the expression of other acquired associations. This is consistent with a substantial body of evidence proposing that altered inhibitory learning may contribute to the development of psychotic symptoms in schizophrenia (Clifton et al., 2017; Holt et al., 2009; Martins Serra et al., 2001; Millan et al., 2012; Pocklington et al., 2015), and reinforces the translational validity of rodent ketamine administration as a model of schizophrenia-like phenotypes.

In addition to NMDA receptor antagonism, ketamine modulates additional neurotransmitter systems in the brain (Kapur and Seeman, 2002; Sleigh et al., 2014; Zorumski et al., 2016), and may induce antidepressant effects (Zanos et al., 2016), differentiating it from other NMDA receptor antagonists and adding uncertainty to the pharmacological origin of its effects. It is informative that NMDA receptor antagonists MK-801, AP5 and PCP have also been reported to impair the extinction and/or latent inhibition of fear memories (Baker and Azorlosa, 1996; Falls et al., 1992; Lee et al., 2006; Lewis and Gould, 2004; Liu et al., 2009; Pollard et al., 2012; Schauz and Koch, 2000; Traverso et al., 2012). Furthermore, D-cycloserine, an NMDA receptor partial agonist, facilitates the extinction of conditioned fear (Bouton et al., 2008; Gabriele and Packard, 2007; Ledgerwood et al., 2003, 2005; Norberg et al., 2008; Richardson, 2004; Walker et al., 2002). It therefore seems plausible that ketamine mediates its effects on the extinction of contextual fear memory *via* NMDA receptor antagonism, although further study of the impact of ketamine’s off-site targets, including the pharmacological activity of its metabolites (Zanos et al., 2016), and its subtype selectivity (Mion and Villevieille, 2013; Zorumski et al., 2016) on inhibitory fear learning is necessary.

Ketamine in healthy volunteers can produce persistent delusions similar to those seen in schizophrenia (Corlett et al., 2007a). The authors suggest that delusions are formed and maintained due to aberrant direct and indirect (*via* altered attribution of salience to stimuli) disruptions in associative learning mechanisms by prediction errors generated by the difference between expectations (“priors”) and current experience (Corlett et al., 2007a, 2016). In support, they show that reactivating fear memories in the presence of ketamine enhances their subsequent expression (Corlett et al., 2013), and that this effect is due in part to an enhancement of reconsolidation processes at retrieval (Honsberger et al., 2015, but see Cassini et al., 2017). Our data is in keeping with this hypothesis such that ketamine, by attenuating extinction, also results in intransient memory that perhaps underpins the characteristic fixity of delusions. We additionally show that ketamine does not alter the consolidation of contextual fear memory, which may indicate a selective effect of ketamine on the distinct associative processes that modulate the expression of previously acquired associations. The retrieval of contextual fear memory in rats trained in the presence of ketamine is only seen when ketamine accompanies retrieval. This may indicate the importance of interceptive stimuli in the recall of ketamine-associated memories. Therefore, during psychotic episodes, a bias may exist towards retrieval of aberrant memories that underpin delusions and which are accompanied by altered associative processes that serve to reinforce them.

The present study highlights a divergence in the effect of pre-training ketamine on excitatory and inhibitory associative learning processes. It also implicates altered extinction, or inhibitory-type associative learning, in the induction of schizophrenia-like behaviour by ketamine. These findings are consistent with impaired extinction learning in schizophrenia (Holt et al., 2009) and parallel the selective impact of schizophrenia-related copy number variants on molecular processes engaged during extinction learning (Clifton et al., 2017). Future research should aim to better understand the molecular processes that set extinction aside from other types of associative learning, with a view to aid the design of novel therapeutics for schizophrenia.

## Supplementary Material

Supplementary material
