# Supplementary material for: The effect of ketamine on the consolidation and extinction of contextual fear memory
Source: J Psychopharmacol. 2018 Jan 17;32(2):156–62. doi: 10.1177/0269881117748903 (PMC5818023; doi:10.1177/0269881117748903)
Supplement: Supplementary material [file JOP748903_Supplementary_Material.pdf]

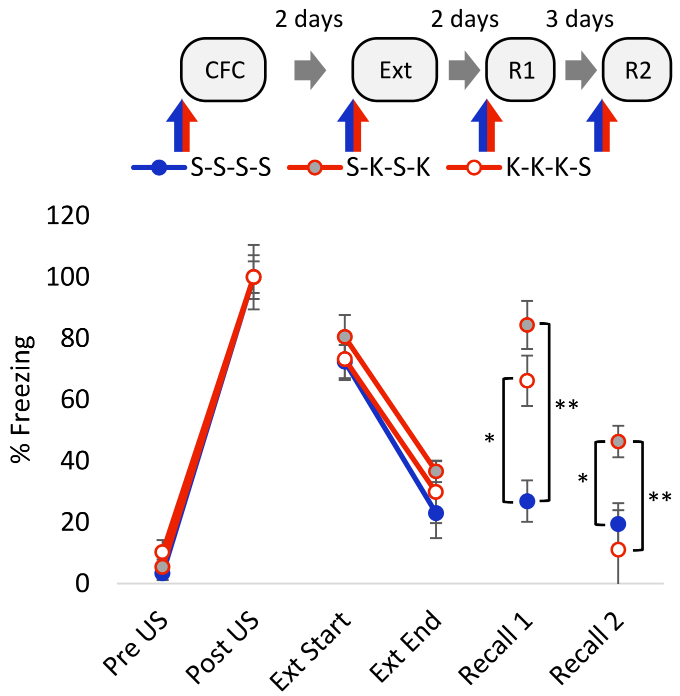

Supplementary Figure 1 The effect of 25 mg/kg ketamine on fear extinction. Experimental groups expanded to show order of treatment. Displayed are the first (Ext Start) and last (Ext End) 2 mins of the extinction trial. 25 mg/kg ketamine or saline vehicle was administered IP 30 min before trials. n=6 per group. In recall trials, ketamine-treated rats received either the same (Recall 1) or different (Recall 2) administration to that received prior to conditioning. Data represented by mean ±SEM of percent freezing response normalised to Post-US. ***P*<0.01, **P*<0.05 in one-way ANOVA with Tukey's multiple comparisons test. CFC, contextual fear conditioning; Ext, Extinction trial; R1, Recall 1; US, unconditioned stimulus; S, saline; K, ketamine.

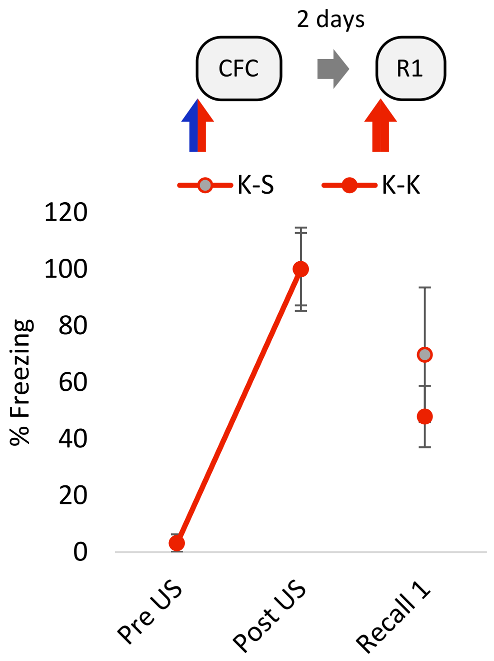

Supplementary Figure 2 8 mg/kg ketamine does not induce a state-dependent contextual fear memory. Ketamine (8 mg/kg) or saline vehicle was administered IP 30 min before conditioning and recall trials. n=4. Data represented by mean ±SEM of percent freezing response normalised to Post-US. CFC, contextual fear conditioning; R1, Recall 1; K, ketamine; S, saline; US, unconditioned stimulus.

Supplementary Table 1 Group size calculations

Effect size	Power	Required N per group
1.5	0.8	9
2.0	0.8	6
2.5	0.8	4
3.0	0.8	4

Predicted effect size (Hedges' *g*; based on previous studies) and target power were used to estimate the required number of subjects (N) per group.

Supplementary Table 2 Contextual fear conditioning experiments freezing data

Group	N	Pre-US (mean±SEM)	Post-US (mean±SEM)	Recall 1 (mean±SEM)	Recall 2 (mean±SEM)	Recall 3 (mean±SEM)
The effect of 25 mg/kg pre-training ketamine on contextual fear conditioning						
S-S	6	1.39±1.39	69.44±10.02	75.00±10.32	52.78±10.90	50.00±9.13
K-K	6	6.94±3.34	63.89±5.12	6.94±3.98	31.94±12.44	18.06±3.98
The effect of 8 mg/kg pre-training ketamine on contextual fear conditioning						
K-S	4	2.08±2.08	62.50±7.98	43.75±16.80	-	-
K-K	4	2.08±2.08	54.17±7.98	27.08±8.59	-	-

Mean ± SEM percent freezing without normalisation to post-US levels.

Supplementary Table 3 Extinction experiments freezing data

Group	N	Pre-US (mean±SEM)	Post-US (mean±SEM)	Ext Start (mean±SEM)	Ext End (mean±SEM)	Recall 1 (mean±SEM)	Recall 2 (mean±SEM)
The effect of 25 mg/kg pre-training ketamine on the extinction of contextual fear memory							
S-S-S-S	6	2.78±1.76	83.33±4.30	59.72±3.98	18.06±5.86	22.22±5.56	15.28±4.52
S-K-S-K	6	4.17±1.86	77.78±5.56	61.11±3.51	29.17±4.69	66.67±8.87	36.11±5.12
K-K-K-S	6	6.94±2.56	63.89±6.69	45.8±4.69	18.06±5.01	40.28±8.45	8.33±6.80
The effect of 8 mg/kg pre-training ketamine on the extinction of contextual fear memory							
S-S	6	1.39±1.39	80.56±7.95	61.11±11.92	20.83±8.54	25.00±8.87	26.39±9.96
S-K	6	1.39±1.39	63.89±5.12	52.78±7.66	12.50±2.85	12.50±4.17	6.94±4.52
K-S	6	1.39±1.39	63.89±6.69	59.72±10.85	16.67±6.80	33.33±9.86	22.22±4.12
K-K	6	4.17±2.85	58.33±5.69	61.11±12.67	20.83±8.81	38.89±10.47	41.67±10.76
The effect of 25 mg/kg post-training ketamine on the extinction of contextual fear memory							
S-S-S	6	2.78±1.76	75.00±3.73	58.33±7.45	20.83±5.16	27.78±6.33	41.67±8.05
S-K-S	6	1.39±1.39	75.00±5.69	58.33±6.45	30.56±3.51	29.17±8.54	26.39±6.60
K-K-S	6	2.78±1.76	66.67±7.45	68.06±11.67	16.67±8.61	26.39±7.27	37.5±5.59

Mean ± SEM percent freezing without normalisation to post-US levels. US, unconditioned stimulus; Ext, extinction trial; S, saline; K, ketamine.
